# Pharmacological Potential and Synthetic Approaches of Imidazo[4,5-*b*]pyridine and Imidazo[4,5-*c*]pyridine Derivatives

**DOI:** 10.3390/molecules22030399

**Published:** 2017-03-04

**Authors:** Malwina Krause, Henryk Foks, Katarzyna Gobis

**Affiliations:** Department of Organic Chemistry, Medical University of Gdańsk, 107 Gen. Hallera Ave., 80-416 Gdańsk, Poland; malwinakrause@gumed.edu.pl (M.K.); hfoks@gumed.edu.pl (H.F.)

**Keywords:** imidazo[4,5-*b*]pyridine, imidazo[4,5-*c*]pyridine, biological activity, synthesis, SAR analysis

## Abstract

The structural resemblance between the fused imidazopyridine heterocyclic ring system and purines has prompted biological investigations to assess their potential therapeutic significance. They are known to play a crucial role in numerous disease conditions. The discovery of their first bioactivity as GABA_A_ receptor positive allosteric modulators divulged their medicinal potential. Proton pump inhibitors, aromatase inhibitors, and NSAIDs were also found in this chemical group. Imidazopyridines have the ability to influence many cellular pathways necessary for the proper functioning of cancerous cells, pathogens, components of the immune system, enzymes involved in carbohydrate metabolism, etc. The collective results of biochemical and biophysical properties foregrounded their medicinal significance in central nervous system, digestive system, cancer, inflammation, etc. In recent years, new preparative methods for the synthesis of imidazopyridines using various catalysts have been described. The present manuscript to the best of our knowledge is the complete compilation on the synthesis and medicinal aspects of imidazo[4,5-*b*]pyridines and imidazo[4,5-*c*]pyridines reported from the year 2000 to date, including structure–activity relationships.

## 1. Introduction

**Figure 1:**
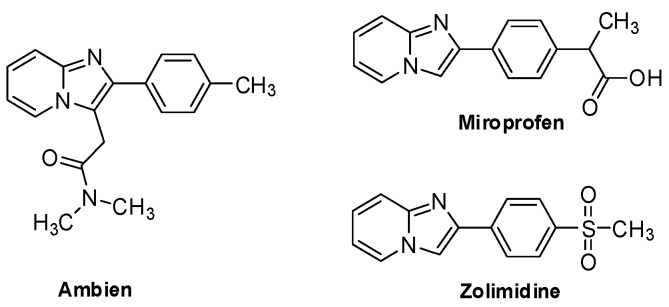
Examples of bioactive agents possessing imidazo[1,2-*a*]pyridine core.

The imidazopyridines comprised an imidazole ring fused with a pyridine moiety. The group contains compounds with different biological activity. In general, they are GABA_A_ receptor agonists [1], however, recently proton pump inhibitors [2], aromatase inhibitors [3], NSAIDs [4] and other classes of drugs in this class have been developed as well. Imidazopyridines consist of various isomeric forms like imidazo[4,5-*b*]pyridines, imidazo[4,5-*c*]pyridines, imidazo[1,5-*a*]pyridines and imidazo[1,2-*a*]pyridines. Among the latter group, one can find the most examples of drugs, such as ambien, miroprofen, and zolimidine (Figure 1).

**Figure 2:**
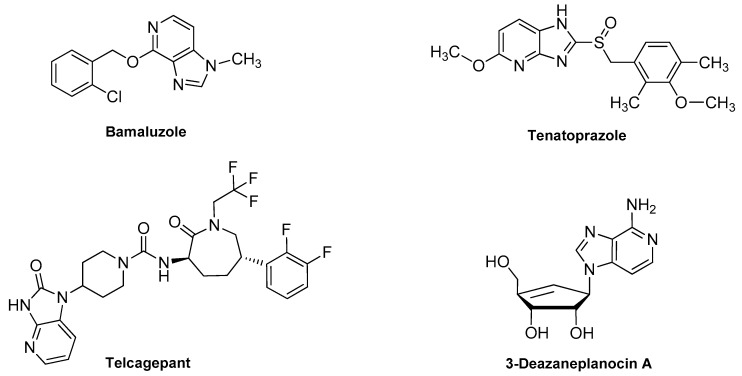
Examples of bioactive agents possessing imidazo[4,5-*b*]pyridine or imidazo[4,5-*c*]pyridine cores.

However, the other two groups seem to also be very potent. In particular, some compounds of the imidazo[4,5-*b*]pyridine and imidazo[4,5-*c*]pyridine core are substances with confirmed activity, and some are at various stages of clinical trials. Bamaluzole with the imidazo[4,5-*c*]pyridine system is a GABA_A_ receptor agonist patented as an anticonvulsant by Merck, but never marketed (Figure 2) [5]. They also discovered telcagepant (MK-0974), a calcitonin gene related peptide receptor antagonist with an imidazo[4,5-*b*]pyridine moiety, which was in clinical trials as a remedy for migraines but its development was terminated in 2009 [6]. Mitsubishi Tanabe Pharma invented tenatoprazole (TU-199) that blocked the gastric proton pump leading to a decline of gastric acid production. This imidazo[4,5-*b*]pyridine is actually under active development by SIDEM (France) [7]. There is also the imidazo[4,5-*c*]pyridine core in the structure of 3-deazaneplanocin A (DZNep) acting as a *S*-adenosyl-l-homocysteine synthesis inhibitor and a histone methyltransferase EZH2 inhibitor, so it can be potentially applied in various types of cancer and Ebola virus disease [8].

The above facts prompted us to take a look at imidazo[4,5-*b*]pyridines and imidazo[4,5-*c*]pyridines as well as their oxygen and sulfur analogues and compare their potency as bioactive agents. The present manuscript to the best of our knowledge is a complete review, including the medicinal aspects, synthetic strategies and structure activity relationship of these heterocyclic groups that is covered from the year 2000 to present.

## 2. Biological Properties of Imidazo[4,5-*b*]pyridines and Imidazo[4,5-*c*]pyridines

### 2.1. Antitumor Activity

In connection with the population growth and an increase in the duration of life we can also observe an escalation of the incidence of non-communicable diseases. Among them cancer is one of the most common causes of death. Imidazopyridines have the ability to influence many cellular pathways necessary for the proper functioning of cancerous cells. The inhibited proteins include, inter alia, matrix metalloproteinase-2, RNA-dependent RNA polymerase, hepatocyte growth factor receptor (c-MET), serine/threonine-protein kinase or cell division cycle 25 phosphatases [9,10,11,12,13,14,15,16,17,18].

**Figure 3:**
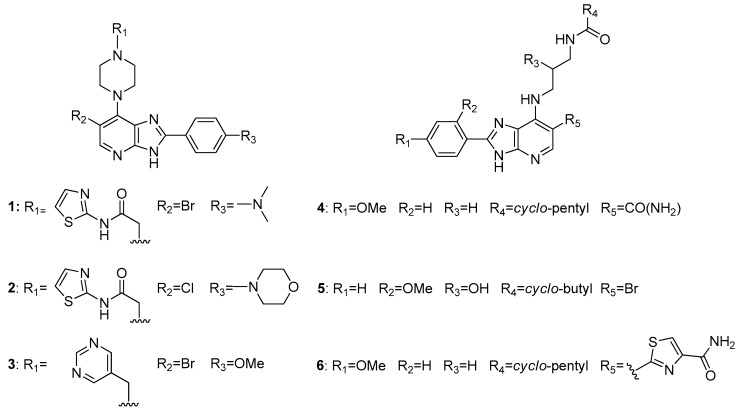
Inhibitors of serine/threonine kinases.

Aurora A, B and C kinases belong to the serine/threonine kinases family. Aurora A kinase (AURKA) is responsible for the proper conduct of mitosis, formation of the mitotic spindle, chromosome segregation and cytokinesis. Scientific research has revealed the overexpression of AURAK in the different types of cancer. There is a probability of connection of AURAK with the carcinogenesis process, therefore, the Aurora A kinase has become a promising molecular target for cancer therapy. Application of imidazo[4,5-*b*]pyridine derivatives **1**–**3** (Figure 3) as inhibitors of AURAKA have been extensively described in the literature [19,20,21].

**Figure 4:**
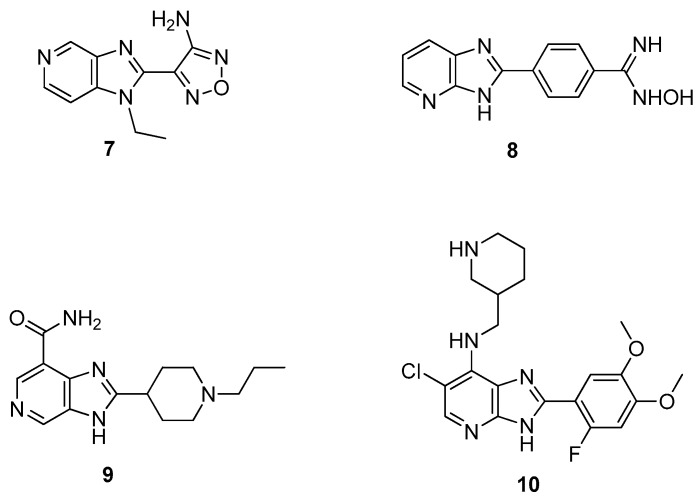
Azolopyridines with antiproliferative activity.

There are also other serine/threonine kinases such as Tank binding kinase 1 (TBK1) and inhibitor of nuclear factor kappa-B kinase subunit epsilon (IKK-ɛ) which are used in the search for antitumor drugs. IKK-ɛ and TBK1 activate nuclear factor kappa-light-chain-enhancer of activated B cells (NF-kappaB) through the process of phosphorylation. NF-kappaB is the transcription factor exhibiting antiapoptoic effects. This results in defective cell survival and tumor development [22,23]. Scientists are trying to develop a new class of drugs that will be exhibit an inhibitory effect on both of the mentioned enzymes. Imidazopyridine derivatives were tested by Wang. Several of the obtained compounds (**4**, **5**, Figure 3) demonstrated inhibitory potency in the range of 0.004–0.046 µM and good selectivity towards cyclin-dependent kinase 2 (CDK2) and Aurora B enzymes [24]. Johanne et al. optimized the structure of compound **4**. Comprehensive modification of the substituent at the C6 position of the imidazopyridine ring has led to obtaining a derivative **6** characterized by higher potency to inhibit both kinases [25]. NF-κB could also be activated by mitogen and stress activated protein kinase 1 (MSK1) protein. Among the MSK1 inhibitors, imidazopyridine (**7**) (Figure 4) with a nanomolar value of IC_50_ (3 nM) has been found [26]. Imidazopyridine derivatives indicated inhibitory impact on inhibitors of apoptosis proteins (IAPs) family and myeloid cell leukemia 1 (Mcl-1) protein [27,28].

Imidazopyridine derivatives have been examined by Puskullu for cytotoxic activity on the human breast adenocarcinoma cell line MCF-7. Compound **8** (Figure 4) with the *N*‑hydroxy- carboximidamide group at the phenyl ring demonstrated the highest cytotoxic activity (IC_50_ 0.082 µM). Substitution of the hydroxycarboximidamide group nitrogen atom by an alkyl chain significantly reduced the activity [29]. In the treatment of breast cancer poly(ADP-ribose) polymerase (PARP) inhibitors were also examined. Zhu et al. obtained the series of imidazo[4,5-*c*]pyridines with moderate to good PARP inhibitory activity. PARP inhibitors increase the sensitivity of tumor cells to chemotherapy. The best potency was demonstrated by compound **9** with an IC_50_ value of 8.6 nM. The obtained compound was also tested in the combination with temozolomide on three human tumor cell lines MDA-MB-468, SW-620 and A549. Research revealed an appreciable increase in the potency of growth inhibition of tumor cells in comparison with the application of only temozolomide [30].

Angiogenesis is the formation process of a blood network necessary for the proper functioning of the body. In healthy subjects, this process is regulated by a plurality of inhibitors and stimulants. Excessive angiogenesis, which is essential for further growth of tumor cells, is observed in the late stages of cancer. The JAK/STAT-3 signaling pathway is involved in the angiogenesis process. Vasbinder attempted to synthesize a selective inhibitor of Januse kinase 1 (JAK-1). Research selected compound **10** with good potency towards JAK-1 (IC_50_ 0.022 µM) and without significant impact on the other isoforms [31]. One of the assignments of the MET pathway in cancer development is the formation of new vessels. Inhibitors of c-MET kinases with an imidazopyridine core may be useful for stopping carcinogenesis [32].

### 2.2. Antimicrobial Activity

In the past few years a large number of bacterial strains resistant to previously used chemotherapeutics agents have emerged. Methicillin resistant *Staphylococcus aureus* (MRSA) and vancomycin-resistant *Enterococcus faecalis* (VRE) cause a serious problems during hospitalization [33,34]. Also, in the case of tuberculosis, an increase in the prevalence of MDR-TB and XDR-TB was observed. Merely 50% of patients with MDR-TB and 26% of patients with XDR-TB completed treatment successfully [35]. The crisis of microbial resistance is a serious public health issue around the world. Obtaining new drugs with new mechanisms of actions is necessary.

**Figure 5:**
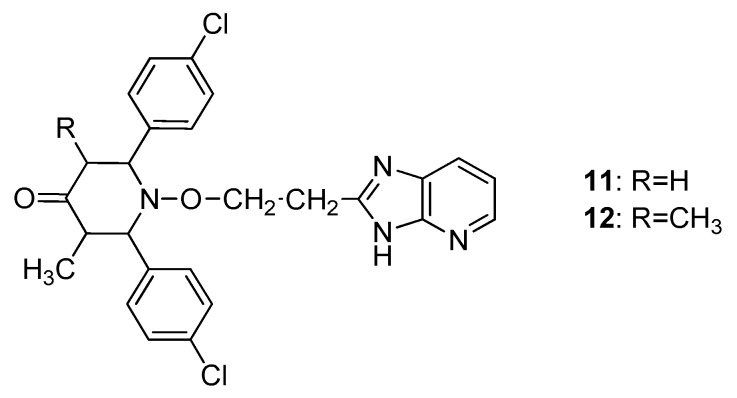
Antibacterial imidazo[4,5-*b*]pyridines.

Arridos et al. combined 2,6-diarylpiperidin-4-one core to the imidazo[4,5-*b*]pyridine ring. Both structures have been widely described in the literature as antimicrobial agents. They observed an increase in activity against Gram positive (*Staphylococcus aureus* and *Bacillus subtilis*) and Gram negative (*Escherichia coli, Pseudomonas aeruginosa* and *Klebsiella pneumonia*) bacteria in the presence of the chlorine atom at the para position of the phenyl groups of compound **11** and **12** (Figure 5). The presence of the methyl group at the C5 position enhanced activity against the tested bacteria strains. The introduction of another methyl group at the C3 position results in a further increment in activity [36].

**Figure 6:**
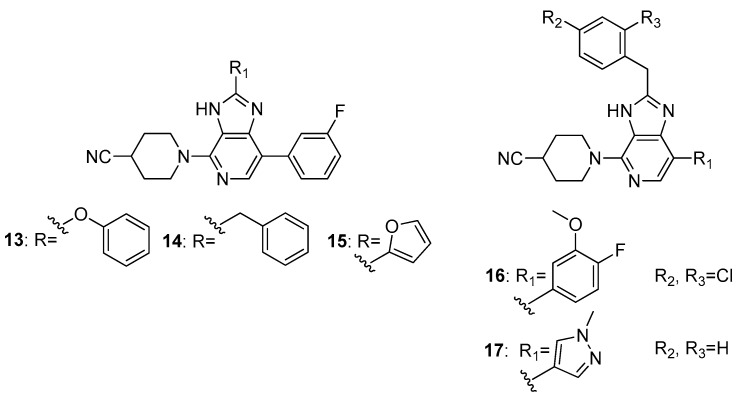
Antifungal imidazo[4,5-*c*]pyridines.

**Figure 7:**
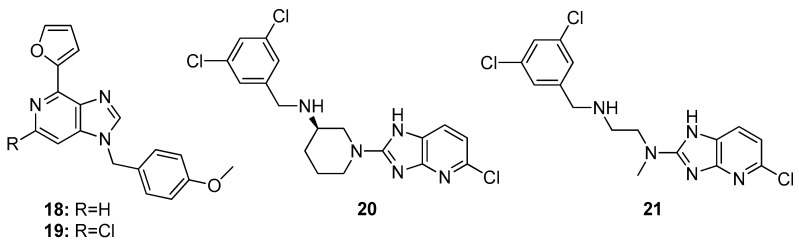
Antimicrobial imidazo[4,5-*b*]pyridines and imidazo[4,5-*c*]pyridines.

Glucosamine-6-phosphate synthase could be a potential molecular target for new imidazo[4,5-*c*]pyridine derivatives in the treatment of fungal infections. This enzyme is responsible for the synthesis of fungal cell walls and regulation of sugar metabolism. The final product of catalysis is uridine diphosphate *N*-acetylglucosamine (UDP-GlcNAc) which is liable for synthesis of cell wall components [37,38]. Compounds **13**–**17** (Figure 6) exhibited good antimicrobial activity in comparison with the reference drugs streptomycin and fluconazole. The molecular docking studies showed that derivatives containing halogen atoms possess good docking energy, oral absorption, skin penetration and membrane permeability [39]. 3-Deazapurines with good tuberculostatic activity were disclosed by Khoje. Compounds **18** and **19** (Figure 7) exhibited activity against *Mycobacterium tuberculosis* with the value of MIC below 1 µM [40].

Certain imidazo[4,5-*b*]pyridine derivatives demonstrated activity inhibiting the growth of *Trypanosoma brucei*. *T brucei* causes in humans the fatal disease African trypanosomiasis [41]. Methionyl-tRNA synthetase of *T. brucei* is an extensively used molecular target in the process of antitrypanosomal drugs development. This enzyme fulfills an essential role in the proper creation of the peptide chain due to the influence on translation [42,43]. Imidazopyridine **20** (Figure 7) exhibited a good inhibitory effect on methionyl-tRNA synthase (IC_50_ < 50 nM, EC_50_ 39 nM). 

The pharmacokinetic parameters have been also examined. Although compound **20** demonstrated remarkable pharmacokinetic parameters after oral administration at 50 mg/kg (Cmax 37.6 µM, and AUC 6223 min·µmol/L) it poorly penetrates into the brain. The brain’s permeability to drugs is extremely important due to the ability of *T. brucei* to cerebrospinal fluid penetration. Structural modifications have led to obtaining derivative **21** with improved brain permeability in mice and inhibition potency (IC_50_ < 50 nM, EC_50_ 22 nM) and moderate pharmacokinetic properties (Cmax 9.7 mM, AUC 952 min·mmol/L) [44].

### 2.3. Anti-Inflammatory

**Figure 8:**
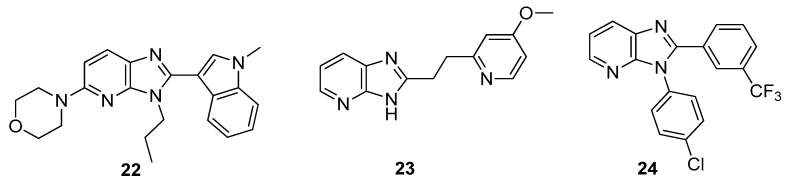
Anti-inflammatory imidazo[4,5-*b*]pyridines.

Inflammation is a defensive reaction of the body caused by chemical, physical or biological agents. The inflammatory response begins with the activation of the immune system and production of inflammatory mediators. Chronic inflammation can lead to harmful effects on the body and the development of other diseases such as cancer and neuropathy. Retinal ischemia is caused by insufficient blood supply in the retina. Sustained ischemia leads to the activation of inflammatory mediators, further degeneration of the retina and impaired vision. Tumor necrosis factor-α (TNF-α), interleukin-6 (IL-6), and adhesion molecules ICAM-1 and VCAM-1 were observed in patients with retinal ischemia [45,46]. Compound **22** (Figure 8) with the imidazo[4,5-*b*]pyridine structure has also been extensively studied as an anti-inflammatory compound in the treatment of retinal ischemia. It has the ability to diminish the *tert*-butyl hydroperoxide-induced inflammatory response in ARPE-19 cells (human retinal pigment epithelial) [47]. Li et al. tested the capability of compound **22** to inhibit the inflammatory reactions associated with obesity. The tested compound affected the activation of transcription factors Nrf2 and NF-κB responsible for regulation of oxidative stress causing arterial injury in the course of obesity [48,49].

Nitric oxide synthase (NOS) is the enzyme that produces nitric oxide(II) which performs many biological functions in human body. One of the inducible NOS isoforms is involved in the immune response. iNOS is activated by proinflamatory factors. However, overexpression of iNOS may lead to adverse reactions such as stroke. The main issue is to achieve a compound which selectively inhibits only iNOS [50,51]. Compound **23** (Figure 8) was characterized by selectivity for iNOS with a pIC_50_ value of 7.09 (nNOS pIC_50_ 4.86; eNOS pIC_50_ 3.95) and good pharmacological parameters. The method of binding compound **23** with l-arginine of isoform iNOS was determined by X-ray crystallography. The methoxy group is essential for selectivity. The 4-methoxy group is nested in a hydrophobic pocket formed by Val346 and Phe363 (murine iNOS numbering) [52].

Cyclooxygenase (COX) is the protein engaged in the formation of prostaglandins, thromboxane and prostacyclin. Cyclooxygenase is part of the inflammatory response. The majority of nonsteroidal anti-inflammatory drugs inhibit the activity of both isoforms COX-1 and COX-2. Numerous side effects are related to the inhibition of COX-1, therefore selective inhibitors of COX-2 are eligible [53,54]. Kirwen et al. investigated the selective COX-2 inhibitors with imidazopyridine moiety. The imidazopyridines obtained contain diaryl pharmacophore which is a key building element of many chemical molecules with anti-inflammatory activity The highest activity and selectivity was demonstrated by compound **24** (COX‑1 IC_50_ 21,8 µmol/L; COX-2 IC_50_ 9,2 µmol/L) (Figure 8) [55].

### 2.4. Antiviral Activity

**Figure 9:**
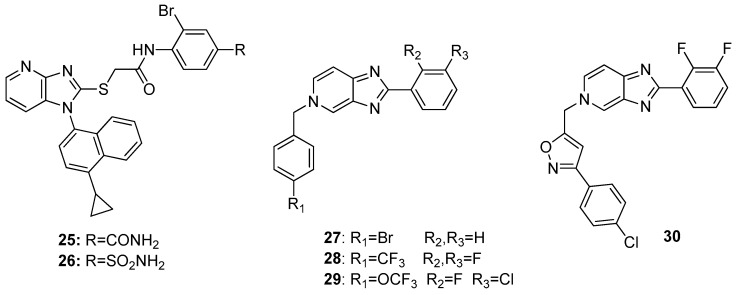
Antiviral imidazo[4,5-*b*]pyridines and imidazo[4,5-*c*]pyridine derivatives.

Viruses have the ability to attack all living organisms, plants, animals and humans. An extremely dangerous phenomenon is their high mutation rate. For this reason, researchers around the world are looking for new antiviral drugs among pyridine derivatives. Human immunodeficiency virus (HIV) is one of the most serious and deadly communicable diseases. Li performed a series of in vitro anti-HIV assays on MT-4 cell culture infected by the HIV-1 IIIB strain, the HIV-1 mutant strain RES056, and the HIV-2 strain ROD. Synthesized compounds demonstrated moderate to good activity. The assays revealed that amide (compound **25**, Figure 9) and sulfamide (compound **26**) groups are preferable in the *para* position of the anilide moiety. The presence of electron-withdrawing group in the *ortho* position of the anilide moiety is likewise relevant for anti-HIV activity. Derivatives **25** and **26** were characterized by higher potency than the reference drugs nevirapine and delaviridine [56].

Puerstinger et al. developed the series of imidazo[4,5-*c*]pyridines and tested them against the *Bovine Viral Diarrhea Virus* (BVDV). As a result of extensive modification, highly active and selective against BVDV molecule **27** (Figure 9) was obtained. A decrease of activity in the presence of a fluorine atom on the phenyl ring located in the 2-position was observed. The presence of large substituents on the benzyl group was associated with the reduction of activity. The received compound interacted with viral RNA-dependent RNA polymerase. The derivatives did not demonstrate activity against the *Hepatitis C Virus* (HCV) which has a similar organizational structure to BVDV. As a second step, compounds selective against HCV were searched. Only a couple of the tested compounds (**28**, **29**) showed selectivity (SI > 595) and high activity (EC_50_ 0.10–0.20 µM) [57,58].

A plurality of modifications in the benzyl group have led to obtaining compound **30** (Figure 9) which is highly potent (EC_50_ 0.004 µM) and selective against HCV. The action mechanism of these compounds is the inhibition of replication of a genotype 2a cell culture infectious HCVcc [59].

### 2.5. Autoimmune Disorders

**Figure 10:**
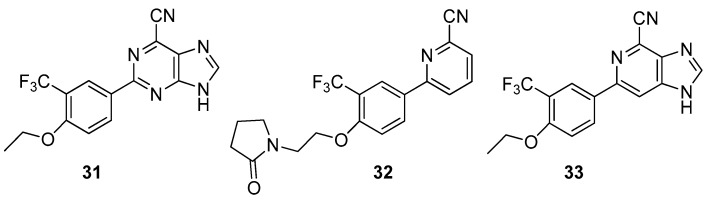
Anti-autoimmune azines **31**–**33**.

Cathepsin S (CTSS) belong to the family of cysteine proteases. Cathepsin S is produced by immune cells presenting antigen, which are activated by inflammatory mediators. The distinctive properties of this protein from other cathepsins are the stability beyond the lysosome and the catalytic activity at neutral pH [60,61]. Inhibition of this enzyme can be effective in the treatment of immune related diseases such as rheumatism or psoriasis. Cai et al. obtained 9*H*-purine-6-carbonitrile **31** (Figure 10) as a good CTSS inhibitor and pyridine-2-carbonitrile **32** which was characterized by the selectivity towards CTSS but with less potency. 

Subsequently, they obtained 1*H*-imidazo[4,5-*c*]pyridine-4-carbonitrile hybrids of these two compounds in order to improve their properties. Among the obtained derivatives compound **33** possessing the ethoxy group showed the highest activity (CTSS IC_50_ 25 nM) while maintaining good selectivity (CTSK IC_50_ 8310 nM). The longer chain or moiety without the oxygen atom indicated a lower activity [62].

### 2.6. Antidiabetic Activity

**Figure 11:**
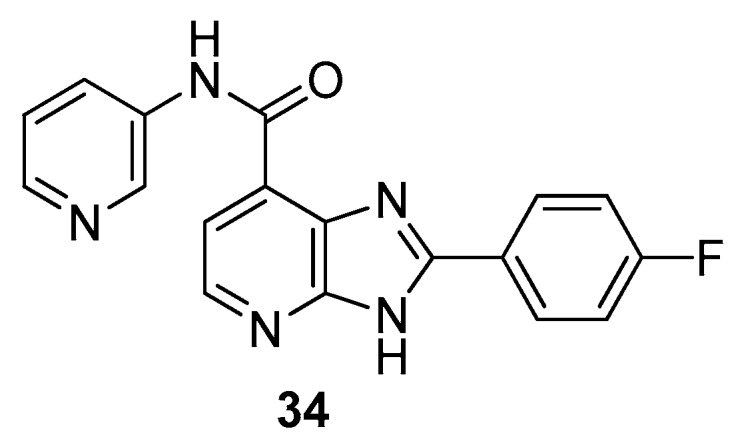
Imidazo[4,5-*b*]pyridine derivative with antidiabetic activity.

Diabetes is one of the most frequently occurring non-communicable diseases. The World Health Organization created the program for the years 2013–2030 which aims to reduces morbidity from diabetes and improvement of pharmacotherapies [63]. Hypoglycemic treatment in the early stages is relatively simple. It requires lifestyle changes and the introduction of monotherapy. Unfortunately, diabetes is a progressive disease and over time it requires the implementation of combination therapy. The most commonly used drugs include sulfonylureas, GLP-1 receptor agonists, PPAR-α agonists, DPP-IV inhibitors and the recently introduced glinides [64,65]. Novel glycogen synthase kinase 3 (GSK-3) inhibitors have been developed. 7-Hydroxybenzimidazole initially obtained by Lee showed good inhibitory activity to the enzyme GSK-3. Physicochemical properties including polar surface area (PSA) have been insufficient. Compounds having the phenol scaffold are metabolized by glucuronidation to ether* O*-glucuronides [66]. Accordingly, the author exchanged the 7-hydroxybenzimidazole moiety for an imidazopyridine which can also be metabolized by glucuronidation. The results revealed that the newly synthesized derivatives have a stronger inhibitory effect on GSK-3 (IC_50_ 1–12 nM) than their 7-hydroxybenzimidazole analogues. X-ray crystallography demonstrated that the imidazopyridine interacted with a carbonyl and an amino group of Val135 as a hydrogen-bonding donor and as a hydrogen-bonding acceptor. In contrast, 7‑hydroxybenzimidazoles interacted with Asp133 and Pro136. Microsomal mouse stability studies were performed for received compounds. Compound **34** (Figure 11) was characterized by good stability (70%), high potency (IC_50_ 8 nM) but also good physicochemical properties such as cLogP (2.73) and a PSA (83.56 Ĺ2) [67].

### 2.7. Miscellaneous Activities

**Figure 12:**
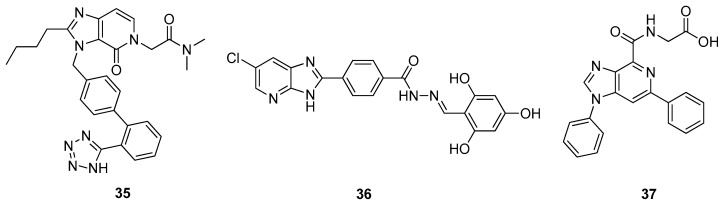
Imidazo[4,5-*b*]pyridine and imidazo[4,5-*c*]pyridine derivatives with a variety of activities.

Sharma et al. reported the series of imidazopyridine derivatives like **35** as angiotensin II receptor antagonists (Figure 12). ARBs act by inhibiting the angiotensin type I receptor thereby preventing the action of angiotensin II and consequently reduce blood pressure [68].

The Maillard reaction is a common reaction occurring between amino acids and reducing sugars. The reaction leads to Amadori products which may accumulate in the human body and cause chronic diseases [69,70]. Following the merger of imidazopyridine and benzohydrazide, compounds with antiglycation and antioxidative potential were obtained. The tendency to an increase in both activities with the increasing number of the hydroxyl group was observed. It is related to high redox potential and the ability to donate electrons. Compound **36** is characterized by the highest antioxidant (EC_50_ 26.12 µM) and an antiglycation (IC_50_ 140.16 µM) activity even higher than for references compounds gallic acid and rutin [71,72].

Frohn reported the synthesis of compound **37** with excellent inhibitory activity towards prolyl hydroxylase domain-2 (PHD2) with the value of IC_50_ 0.003 µM. PHD2 is critical for the regulation of hypoxia-inducible factor (HIF), whereas the hypoxia-inducible factor is engaged in the process of erythropoiesis [73,74].

## 3. Synthetic Routes to Imidazo[4,5-*b*]pyridines and Imidazo[4,5-*c*]pyridines

While many strategies are available for imidazo[4,5-*b*]pyridine and imidazo[4,5-*c*]pyridine synthesis, the most popular approaches involve both condensation–dehydration reactions of pyridine-2,3-diamine or pyridine-3,4-diamine with carboxylic acids (or their equivalents) and condensation with aldehydes under oxidative conditions. Carboxylic acids or their equivalents (nitriles, amidates, and orthoesters) are usually reacted under strongly acidic or harsh dehydrating conditions that often require high temperatures or the use of reagents such as phosphorus anhydride. The reaction can also be performed stepwise via intermediate amides. In the case of aldehydes, the reaction proceeds through an imidazolidine-pyridine, and this requires an oxidative step for conversion to the corresponding imidazopyridine. Recently, a number of modifications of known methods were described that may significantly accelerate the reactions and increase their yields.

**Scheme 1:**
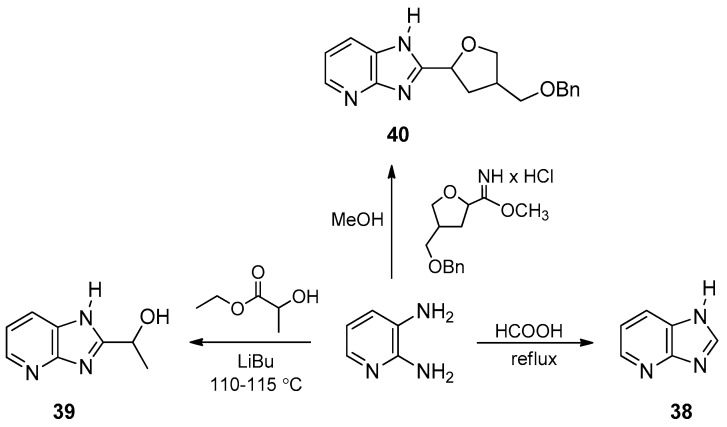
Synthesis of imidazo[4,5-*b*]pyridines from 2,3-diaminopyridine and carboxylic acids or their functional derivatives.

According to the classical method of benzimidazole synthesis, Dymińska et al. proposed the synthesis of imidazo[4,5-*b*]pyridine **38** from 2,3-diaminopyridine and formic acid (Scheme 1) [75]. The first successful lithium bromide mediated solvent free condensation of arylenediamine and esters to obtain 2-substituted imidazopyridines (e.g., **39**) in good to excellent yields at 110–115 °C was described by Dekhane et al. [76]. Mladenova performed the reaction of methyl 4-((benzyloxy)methyl)tetrahydrofuran-2-carbimidate hydrochloride–*cis* or *trans* with 2,3-diaminopyridine in MeOH resulting in the corresponding 1*H*-imidazo[4,5-*b*]pyridine **40**, *cis* or *trans* in a yield of 40% [77].

**Scheme 2:**
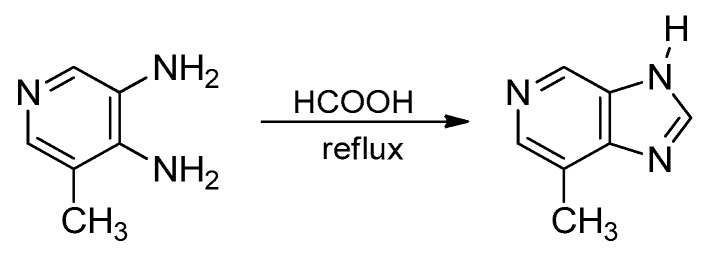
Synthesis of methyl-substituted imidazo[4,5-*c*]pyridines.

Dymińska also obtained 7-methyl-3*H*-imidazo[4,5-*c*]pyridine from 5-methyl-3,4-diamino-pyridine in 100% formic acid (Scheme 2). The reaction mixture was boiled under reflux for 6 h and was recommended as a method for the preparation of derivatives substituted with a methyl group at various positions of the pyridine ring [78].

**Scheme 3:**
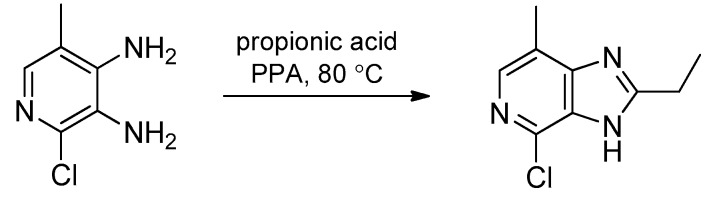
Synthesis of functionalized imidazo[4,5-*c*]pyridine.

A typical reaction for the preparation of imidazopyridines seems to be the synthesis from carboxylic acid and 2,3-diaminepyridine or 3,4-diaminopyridine in polyphosphoric acid (PPA) as a dehydrating agent at an elevated temperature (Scheme 3). The method is simple, convenient and rather good yielded (~75%), particularly when microwave irradiation is used [79].

**Scheme 4:**
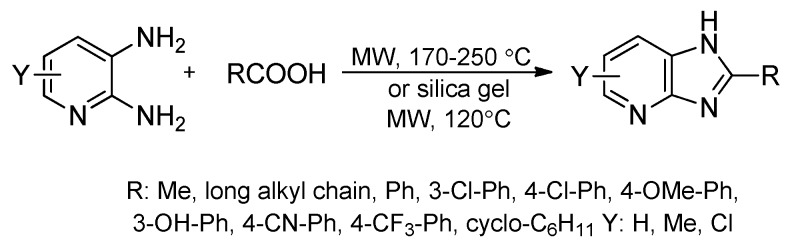
Microwave assisted synthesis of imidazo[4,5-*b*]pyridines.

The condensation reaction of 2-amino-3-hydroxypyridine with different carboxylic acids by microwave-assisted heating is a fast method for producing libraries based on fused 2-substituted imidazo[4,5-*b*]pyridines in moderate to good yields (Scheme 4). The best results (yields 71%–92%) were obtained by using silica gel as a support and equimolecular quantities of the both reactants at 100 W [80,81].

**Scheme 5:**
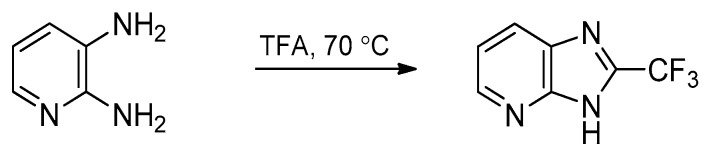
Synthesis of fluoroalkyl-imidazo[4,5-*b*]pyridines.

Rene et al. reported an efficient one-step route to fluoroalkyl-azabenzimidazoles via the condensation of fluorinated carboxylic acids and pyridinediamines (Scheme 5). This method is high-yielding (54%–99%) with a broad scope and is operationally simple with potential application to parallel synthesis [82].

**Scheme 6:**
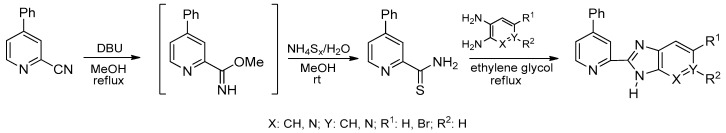
Imidazopyridine synthesis from carbonitrile via picolinothioamide.

Imidazo[4,5-*b*]pyridine and imidazo[4,5-*c*]pyridine derivatives were also synthesized from 4-phenylpicolinothioamide (Scheme 6), which was obtained from starting carbonitrile via methyl carbimidate in the reaction with DBU and ammonium polysulfide. Then, upon treatment of appropriate diamines in ethylene glycol, 4-phenylpicolinothioamide gave the corresponding imidazopyridines [83].

**Scheme 7:**
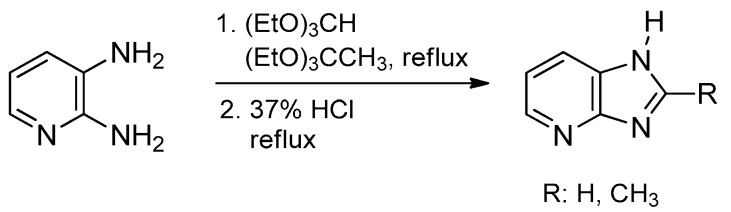
Imidazopyridine synthesis using orthoesters.

The cyclization to the imidazo[4,5-*b*]pyridine ring system was also effected by refluxing 2,3-diaminopyridine in triethyl orthoformate or triethyl orthoacetate followed by chlorohydric acid treatment to obtain, respectively, imidazo[4,5-*b*]pyridine its 2-methyl derivative in yield 83% and 78% (Scheme 7) [84].

**Scheme 8:**
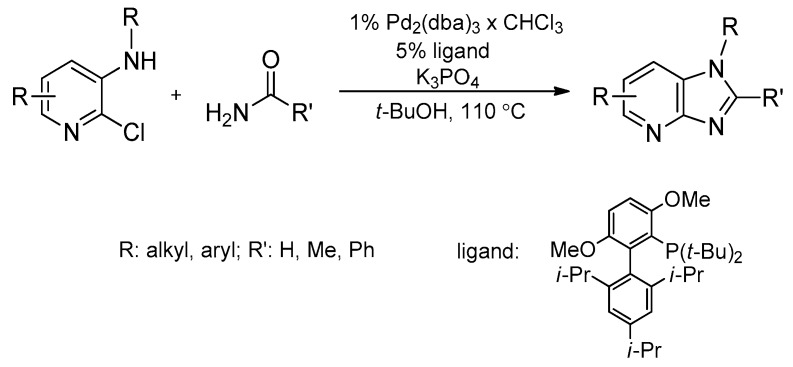
Pd-catalyzed amide coupling reaction produces imidazo[4,5-*b*]pyridines.

A facile synthesis of imidazo[4,5-*b*]pyridines was described by Rosenberg et al. using a Pd-catalyzed amide coupling reaction. 3-Alkyl and 3-arylamino-2-chloropyridines reacted with simple primary amides in good yields (51%–99%) when refluxing in *t*-butanol in the presence of tris(dibenzylideneacetone)dipalladium(0)-chloroform adduct, di-tert-butyl(2′,4′,6′-triisopropyl-3,6-dimethoxy-[1,1′-biphenyl]-2-yl)phosphine as a ligand and potassium phosphate (Scheme 8). That reaction provided quick access to products with substitution at N1 and C2 [85].

**Scheme 9:**
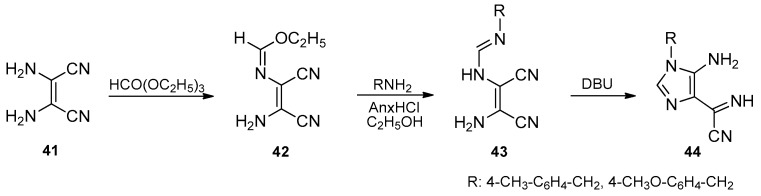
Synthesis of 5-amino-1-(substituted benzyl)-4-cyanoformimidoyl imidazole derivative as an interediate for imidazole[4,5-*b*]pyridines.

Al-duaij et al. [86] described an interesting method of imidazo[4,5-*b*]pyridine synthesis using malononitrile. First, heating diaminomaleonitrile **41** with triethyl orthoformate in dioxane afforded ethyl [2-amino-1,2-dicyanovinyl]imidoformate **42**. Treatment of **42** with the appropriate amines, namely 4-methoxybenzylamine and 4-methylbenzylamine catalyzed by aniline hydrochloride, formed (substituted benzyl)-*N*-(2-amino-1,2-dicyanovinyl)formimidine **43** which underwent intramolecular cyclization in the presence of 1,8-diazabicyclo(5.4.0)undec-7-ene (DBU) to form 5-amino-1-(substituted benzyl)-4-cyanoformimidoyl imidazole derivative **44** (Scheme 9).

**Scheme 10:**
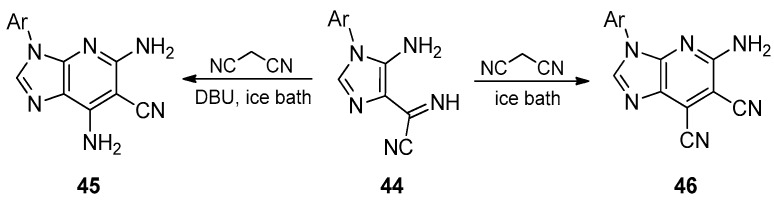
Synthesis of functionalized imidazo[4,5-*b*]pyridines from 1-aryl-5-amino-4-(cyanoformimidoyl)imidazoles.

Next 1-aryl-5-amino-4-(cyanoformimidoyl)imidazoles **44** were reacted with malononitrile under mild experimental conditions and led to 3-aryl-5,7-diamino-6-cyano-3*H*-imidazo[4,5-*b*]pyridines **45**, when the reaction was carried out in the presence of DBU, or to 3-aryl-5-amino-6,7-dicyano-3*H*-imidazo[4,5-*b*]pyridines **46**, in its absence. Both reactions evolved from the adduct formed by a nucleophilic attack of the malononitrile anion to the carbon of the cyanoformimidoyl substituent (Scheme 10) [86,87].

**Scheme 11:**
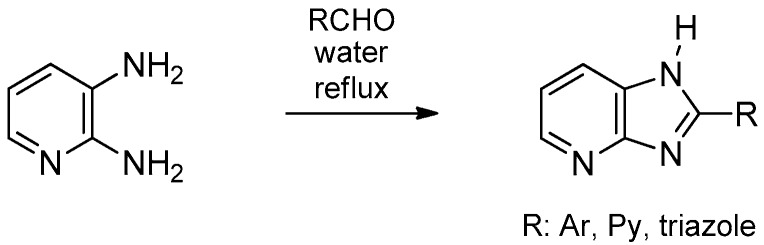
Classical synthesis from 2,3-diaminopyridine and aldehydes.

A new environmentally-benign, convenient, and facile methodology for the synthesis of 2-substituted-1*H*-imidazo[4,5-*b*]pyridine was elaborated by Kale et al. [88]. The reaction of 2,3-diaminopyridine with substituted aryl aldehydes in water under thermal conditions without the use of any oxidative reagent has been studied. The reaction has yielded 1*H*-imidazo[4,5-*b*]pyridine derivatives (Scheme 11) by an air oxidative cyclocondensation reaction in one step in an excellent yield (83%–87%). Ivanova et al. described similar preparation of substituted 2-(1,2,4-triazol-3-yl)imidazopyridines from diaminopyridines and triazole aldehydes. In that case also, good yields were obtained 37%–71% [89].

**Scheme 12:**
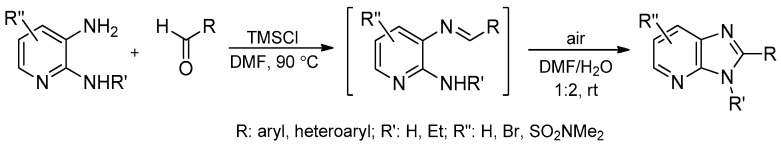
Modification with chlorotrimethylsilane as apromoter and water-acceptor.

A set of 3*H*-imidazo[4,5-*b*]pyridines was also readily prepared from (hetero)aromatic *ortho*-diamines and aldehydes using chlorotrimethylsilane in DMF as a promoter and water-acceptor agent, followed by oxidation with air oxygen (Scheme 12). The authors obtained products with very good yields 79%–80% [90].

**Scheme 13:**
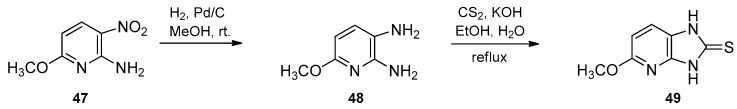
Synthesis of imidazo[4,5-*b*]pyridine-2-thione.

Reduction of 6-methoxy-3-nitropyridin-2-amine **47** was achieved catalytically (using Pd/C as a catalyst under hydrogen gas) to give the compound 6-methoxypyridine-2,3-diamine (**48**). This diamino analog was instable and immediately used in the next step reaction without further purification (Scheme 13). Subsequent imidazole ring compound **49** was formed from compound **48** by treating with CS_2_ and KOH in 63% yield [91].

**Scheme 14:**
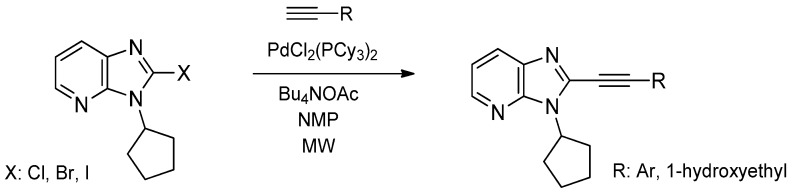
Synthesis of 2-halo-3-alkyl imidazo[4,5-*b*]pyridines.

Enhancing the reactivity of the catalytic system by using a palladium catalyst with sterically demanding and electron rich ligands attached to it has often been shown as an appropriate way of performing the copper-free Sonogashira reaction. Sajith et al. [92] reported PdCl_2_(PCy_3_)_2_ (Scheme 14) as an efficient catalyst for the copper and amine-free Sonogashira cross coupling reactions of 2-halo-3-alkyl imidazo[4,5-*b*]pyridines (I, Br, Cl) using tetrabutyl ammonium acetate as an activator in *N*-methylpyrrolidone (NMP) under microwave enhanced conditions (yields 70%–95%).

**Scheme 15:**
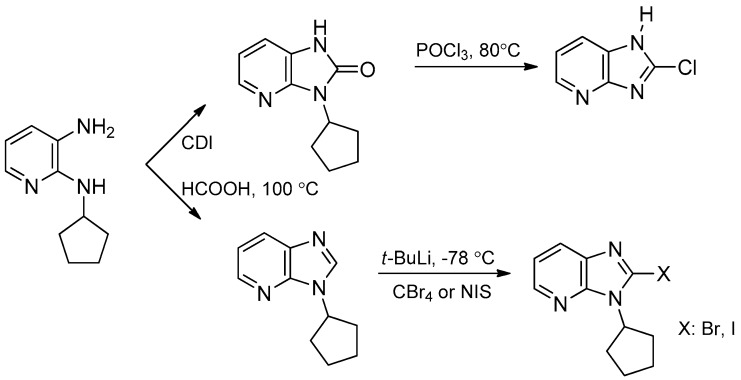
Synthesis of 2-halo-imidazo[4,5-*b*]pyridines.

The same authors described the synthesis of imidazo[4,5-*b*]pyridines in the reaction with 1,1′-carbonyldiimidazole (CDI) and then with phosphoryl chloride or with formic acid followed by halogenation with carbon tetrabromide or *N*-iodosuccinimide (NIS) (Scheme 15) [93].

**Scheme 16:**
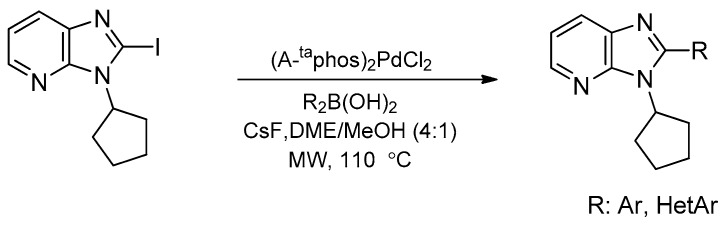
Synthesis of 3-substituted 2-aryl/heteroaryl imidazo[4,5-*b*]pyridines.

They also proposed a modified approach for the synthesis of 3-substituted 2-aryl/heteroaryl imidazo[4,5-*b*]pyridines with excellent yields. The method utilizing palladium catalysed cross-coupling reactions under microwave enhanced conditions. Utilization of (A-^ta^phos)_2_PdCl_2_-catalysed Suzuki-Miyayura cross-coupling reactions enables rapid derivatization of this imidazo[4,5-*b*]pyridine pharmaceutically relevant core (Scheme 16). This catalytic system is compatible with a broad spectrum of arylboronic acids electron rich, electron poor, and heteroarylboronic acids [92]. In another paper the same authors reported the regioisomeric synthesis of fully decorated imidazopyridines employing a C-H activation protocol with a wide range of aryl/heteroaryl/alkyl boronic acids in aqueous DMF. The use of a copper catalyst and bathophenanthroline as a ligand were found to be instrumental in driving these reactions to completion. The optimized protocol was further extended to alkyl/aryl/heteroaryl potassium organotrifluoroborates [94].

**Scheme 17:**
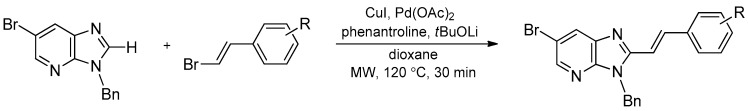
Synthesis of 2-styrene-imidazo[4,5-*b*]pyridines.

Baladi et al. described the first C-2 direct alkenylation of the valuable 3*H*-imidazo[4,5-*b*]pyridine promoted by microwave-assisted Pd/Cu co-catalysis. By using Pd(OAc)_2_, CuI, phenanthroline, and *t*BuOLi in dioxane under microwave irradiation conditions at 120 °C for 30 min, both electron-rich and electron-deficient β-bromostyrenes reacted at the C-2 position of imidazo[4,5-*b*]pyridine in moderate to good yields (Scheme 17). A variety of functional groups including halides, acetals, ethers and cyano moieties were well tolerated under the reaction conditions; and more importantly, such groups can be used for further chemical transformations [95].

**Scheme 18:**
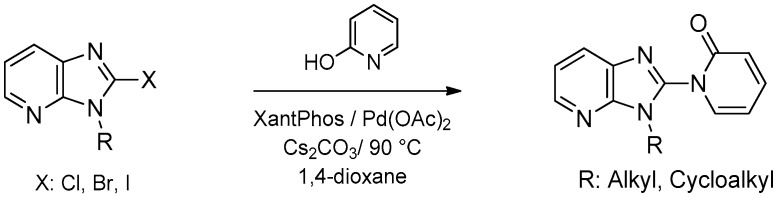
Fuctionalization of imidazo[4,5-*b*]pyridines at the C-2 position using 2-hydroxypyridine with XantPhos and Pd(OAc)_2_.

A rapid and facile access to C2-substituted imidazo[4,5-*b*]pyridine analogues utilizing palladium mediated Buchwald–Hartwig cross-coupling reactions has been reported by Khader et al. [96]. The use of enolizable heterocycles as cross-coupling partners resulted in a wide range of imidazo[4,5-*b*]pyridine analogues which are prone to have medicinal relevance. XantPhos (4,5-bis(diphenylphosphino)-9,9-dimethylxanthene) and Pd(OAc)_2_ were found to be more effective for the coupling of 2-halo imidazo[4,5-*b*]pyridines with pyridone nucleophiles (Scheme 18). A regioselective approach for the synthesis of 2-substituted 3*H*-imidazo[4,5-*b*]pyridine and 1*H*-imidazo[4,5-*b*]pyridine has been also reported. The authors obtained products with yields from medium to excellent (49%–95%). The overall efficiency of a cross-coupling process is significantly affected by the structure of the ligand (BINAP, XantPhos) and the catalyst. Therefore, the use of a ligand with appropriate steric and electronic properties is very crucial in dealing with problematic and specific substrates in this area.

**Scheme 19:**
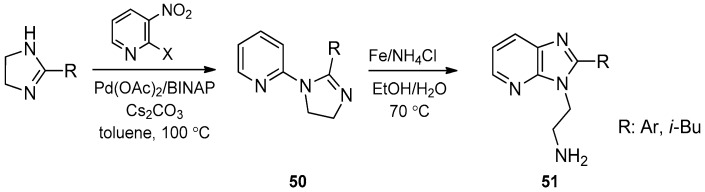
Pd-catalyzed (hetero)arylation of 2-imidazolines.

A facile rearrangement of *N*-(hetero)aryl 2-imidazolines into diversely substituted imidazo[4,5-*b*]pyridines, under Bechamp reduction conditions has been discovered by Mujumdar et al. [97]. Combined with the earlier reported protocol for Pd-catalyzed (hetero)arylation of 2-imidazolines, it provides a simple two-step access to a range of compounds based on this core. The method uses the Pd(OAc)_2_/BINAP catalytic system in the first step to the imidazoline structure **50**, which when subjected to the modified Bechamp reduction conditions (Fe/NH_4_Cl in aqueous EtOH), converted to a more polar imidazopyridine **51** (Scheme 19).

**Scheme 20:**
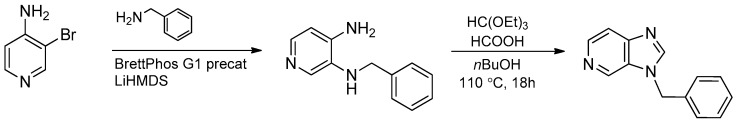
C-N coupling of 4-amino-3-bromopyridine with benzylamine.

A practical strategy for the preparation of imidazopyridine derivatives from *ortho*-haloaminopyridines utilizing a two-step C-N coupling/cyclization reaction sequence has been developed by Li et al. [98]. Their procedure provides rapid and efficient access to many medicinally interesting imidazopyridine compounds and related imidazopyrazine/purine heterocycles. They began the investigation by screening conditions for C-N coupling of 4-amino-3-bromopyridine with benzylamine (Scheme 20). The best catalyst and base were BrettPhos and LiHMDS (82% yield). Alternative catalysts (such as *t*BuXPhos, XPhos, RuPhos or XantPhos) did not afford the desired product in useful yield. Surprisingly, the BrettPhos G1 precatalyst outperformed the BrettPhos G3 precatalyst (45% yield) and Pd(OAc)_2_/BrettPhos (12% yield). The choice of LiHMDS as a base was found to be critical for achieving high yield while none of the other bases (NaHMDS, KHMDS, NaO*t*Bu, LiO*t*Bu, Cs_2_CO_3_) examined gave more than a 35% yield. Additional variables including solvent, concentration, catalyst loading, equivalents of base, and temperatures were also examined. They concluded the following reaction conditions as optimal for the C-N coupling: BrettPhos G1 precatalyst (6 mol%), LiHMDS (2.5 equiv), THF (0.4 M), 40 °C. In addition, we also examined 4-amino-3-iodopyridine and 4-amino-3-chloropyridine as the halide coupling partner and found that the former showed comparable reactivity (85% yield) while the chloride had inferior performance (47% yield).

**Scheme 21:**
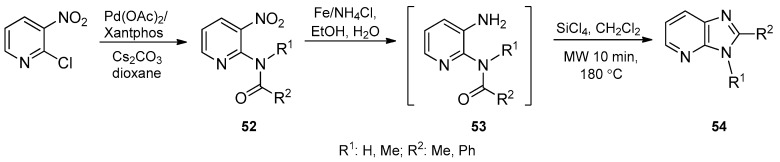
Synthesis of differently substituted imidazo[4,5-*b*]pyridines from 2-chloro-3-nitropyridine.

Salome et al. has presented a very straightforward method for preparing differently substituted imidazo[4,5-*b*]pyridines. The target compounds mainly result from direct amidation of the highly electrophilic 2-chloro-3-nitropyridine with various amides including primary, secondary and cyclic amides using Pd-coupling reactions (Scheme 21). Despite the poor nucleophilic character of amides, when reacted with aryl halides, the reaction could be extended to sulfonamides, carbamates and ureas by means of XanthPhos as a ligand and Cs_2_CO_3_ as the base in dioxane in the presence of Pd(OAc)_2_ or Pd_2_(dba)_3_. The nitro intermediates **52** were quantitatively reduced by means of iron in the presence of ammonium chloride in a mixture of ethanol and water. The resulting crude amino intermediate **53** was further submitted to cyclization using SiCl_4_ as an efficient Lewis catalyst. The reaction can be performed 10 min after exposure to microwave irradiation at 180 °C. The overall yields (reduction and cyclization) were satisfactory to excellent (55%–90%) [99].

**Scheme 22:**
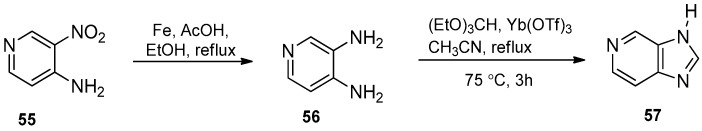
Synthesis of imidazo[4,5-*c*]pyridine from 3,4-diaminopyridine and orthoformate by c ytterbium triflate catalyzed condensation.

The two-step procedure using Fe and acetic acid in ethanol to reduce 3-nitropyridin-4-amine **55** (Scheme 22) followed by Ytterbium triflate catalyzed condensation with triethyl orthoformate to imidazo[4,5-*c*]pyridine **57** by 3,4-diaminopyridine **56** was reported by Wang et al. [100]. The optimum stoichiometry of each component is highlighted and the broad utility was demonstrated with high compatibility to numerous functional groups. The use of the optimal conditions resulted in obtaining the desired products in yields of 32%–99%.

**Scheme 23:**
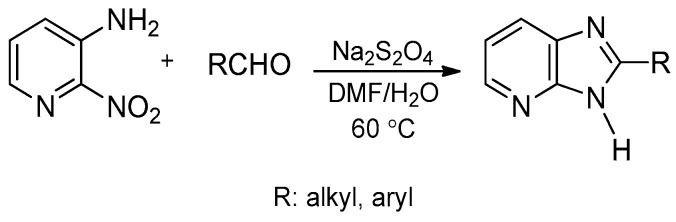
Synthesis of 3*H*-imidazo[4,5-*b*]pyridines from aldehydes and 2-nitro-3-aminopyridine.

Harer et al. discovered a one-step synthesis of 3*H*-imidazo[4,5-*b*]pyridines from aldehydes and 2-nitro-3-aminopyridine through reductive cyclization using Na_2_S_2_O_4_ [101]. Aqueous paste of Na_2_S_2_O_4_ was prepared as 1M in H_2_O and added in 3 equivalent proportions to the reaction mixture (Scheme 23).

**Scheme 24:**
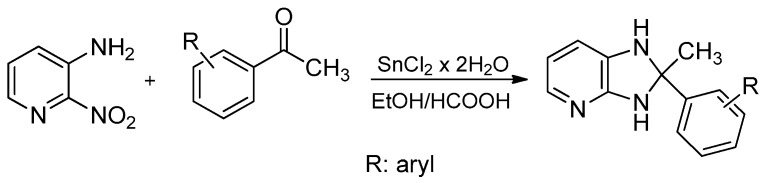
Synthesis of 1*H*-imidazo[4,5-*b*]pyridines from ketones and 2-nitro-3-aminopyridine.

The second reaction for one-step synthesis of 1*H*-imidazo[4,5-*b*]pyridines was obtained from ketones and 2-nitro-3-aminopyridine through reductive cyclization using SnCl_2_×2H_2_O as a reductive catalyst. Imidazopyridine scaffolds were visited after treatment of the substituted acetophenones and 2-nitro-3-aminopyridine with the addition of SnCl_2_×H_2_O in the presence of formic acid (Scheme 24). It is presumably formed through formylation of the aniline nitrogen, nitro reduction and cyclization. Formylation of the aniline nitrogen is believed to assist nitro reduction [101].

**Scheme 25:**
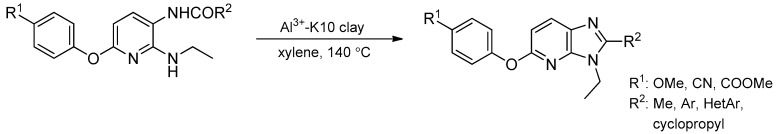
Intramolecular cyclization using Al^3+^-exchanged on K10 montmorillonite clay.

A series of imidazopyridine derivatives has been synthesized efficiently via intramolecular cyclization in excellent yields using Al^3+^-exchanged on K10 montmorillonite clay (Al^3+^-K10 clay) as a reusable heterogeneous catalyst by Suresh et al. [102]. The authors report was the first to utilize Al^3+^-K10 as a catalyst for imidazopyridine synthesis (Scheme 25). Many functional groups were tolerated during the synthesis of targeted compounds and the yields obtained were excellent (80%–93%). The catalyst was reused at least five times with a slight decrease in the yield. This catalyst was environmentally benign, cost-effective, and also provided other advantages such as nontoxicity, operational/experimental simplicity, and mild reaction conditions.

**Scheme 26:**
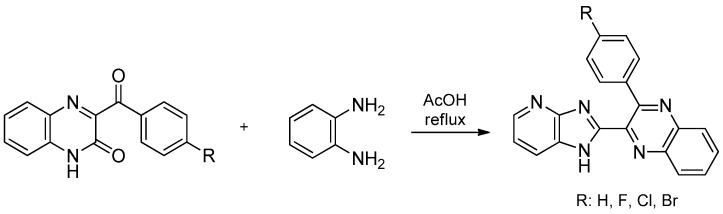
Synthesis of 1*H*-imidazo[4,5-*b*]pyridines from quinoxalinones and their aza-analogues.

A one-step versatile method for the synthesis of 1*H*-imidazo[4,5-*b*]pyridines (Scheme 26) from quinoxalinones and their aza-analogues has been developed on the basis of the novel ring contractions of 3-aroyl-quinoxalinones and their aza-analogues with 1,2-arylenediamines in boiling acetic acid solution with rather good yields of 41%–84% [103].

**Scheme 27:**
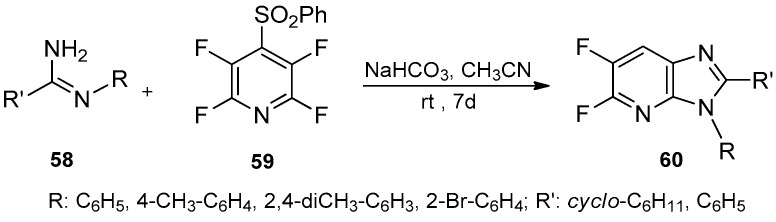
Synthesis of 5,6-difluoro-imidazo[4,5-*b*]pyridines from *N*-aryl amidines and 4-phenylsulfonyl tetrafluoropyridine.

The reactions between *N*-aryl amidines **58** and 4-phenylsulfonyl tetrafluoropyridine **59** were reported by Poorfreidoni et al. [104]. Reactants were stirred at room temperature in the presence of NaHCO_3_ in acetonitrile and corresponding polyfunctional heteroaromatic imidazopyridine systems **60** via an intramolecular nucleophilic aromatic substitution process (Scheme 27). The products’ structure was the result of substitution at the 2-position of the pyridine ring by the amidine secondary nitrogen, followed by intramolecular ring closure at the geometrically accessible 3-position of the pyridine ring by the amidine primary nitrogen.

**Scheme 28:**
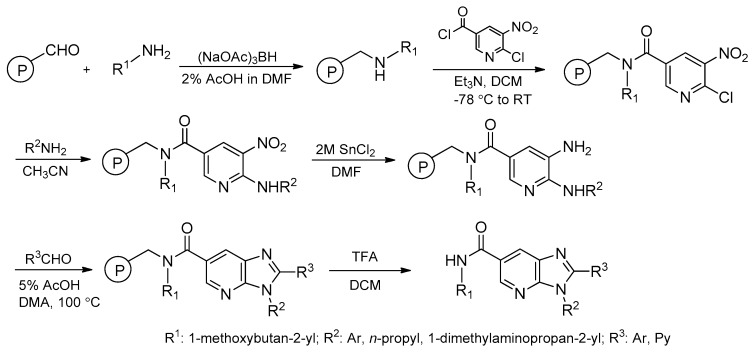
The solid-phase synthesis of 1,2,5-substituted 7-azabenzimidazole derivatives.

Recently, methods for the use of solid-phase to obtain imidazopyridines have been described. A novel route for the solid-phase synthesis of 1,2,5-substituted 7-azabenzimidazole derivatives has been developed by Farrant et al. (Scheme 28) [105]. In this method primary amines are attached to an aldehyde resin then coupled to 6-chloro-5-nitro-nicotinyl chloride. Subsequent alkylation with amines, reduction of the nitro group and cyclization with aldehydes gives 1,2,5-substituted 7-azabenzimidazole derivatives with yields of 50%–94%.

## 4. Conclusions

Numerous compounds derived from the imidazopyridine nucleus are used in the clinic for the treatment of many diseases. Most of them are imidazo[1,2-*a*]pyridine derivatives. However, imidazo[4,5-*b*]pyridines and imidazo[4,5-*c*]pyridines, despite the similar activity, exhaustive and target based research on development of many compounds as antitumor, antimicrobial, anti-inflammatory, antiviral, immunomodulatory, antidiabetic modulators have not made their way to the market and clinic. This is probably due to lack of a comprehensive compilation of various research reports in each activity capable of providing insights into the SAR of the compounds. The present review covering more than 100 the most recent references on activity and chemical synthesis is expected to provide a closer look at the imidazo[4,5-*b*]pyridine and imidazo[4,5-*c*]pyridine-derived compounds for a versatile and target oriented information to develop clinically viable molecules.
